# Integrative bioinformatics analysis to identify novel biomarkers associated with non-obstructive azoospermia

**DOI:** 10.3389/fimmu.2023.1088261

**Published:** 2023-03-08

**Authors:** Yucheng Zhong, Jun Zhao, Hao Deng, Yaqin Wu, Li Zhu, Meiqiong Yang, Qianru Liu, Guoqun Luo, Wenmin Ma, Huan Li

**Affiliations:** ^1^Assisted Reproductive Technology Center, Southern Medical University Affiliated Maternal and Child Health Hospital of Foshan, Foshan, Guangdong, China; ^2^Assist Reproductive Medical Center, Zhaoqing West River Hospital, Zhaoqing, Guangdong, China

**Keywords:** azoospermia, autophagy, hub gene, immune, biomarkers

## Abstract

**Aim:**

This study aimed to identify autophagy-related genes (ARGs) associated with non-obstructive azoospermia and explore the underlying molecular mechanisms.

**Methods:**

Two datasets associated with azoospermia were downloaded from the Gene Expression Omnibus database, and ARGs were obtained from the Human Autophagy-dedicated Database. Autophagy-related differentially expressed genes were identified in the azoospermia and control groups. These genes were subjected to Gene Ontology and Kyoto Encyclopedia of Genes and Genomes, protein–protein interaction (PPI) network, and functional similarity analyses. After identifying the hub genes, immune infiltration and hub gene–RNA-binding protein (RBP)–transcription factor (TF)–miRNA–drug interactions were analyzed.

**Results:**

A total 46 differentially expressed ARGs were identified between the azoospermia and control groups. These genes were enriched in autophagy-associated functions and pathways. Eight hub genes were selected from the PPI network. Functional similarity analysis revealed that *HSPA5* may play a key role in azoospermia. Immune cell infiltration analysis revealed that activated dendritic cells were significantly decreased in the azoospermia group compared to those in the control groups. Hub genes, especially *ATG3*, *KIAA0652*, *MAPK1*, and *EGFR* were strongly correlated with immune cell infiltration. Finally, a hub gene–miRNA–TF–RBP–drug network was constructed.

**Conclusion:**

The eight hub genes, including *EGFR*, *HSPA5*, *ATG3*, *KIAA0652*, and *MAPK1*, may serve as biomarkers for the diagnosis and treatment of azoospermia. The study findings suggest potential targets and mechanisms for the occurrence and development of this disease.

## 1 Introduction

Azoospermia is a reproductive disorder frequently observed in male infertility. Typically, it is classified as either obstructive or non-obstructive azoospermia (NOA) (1). Most patients with azoospermia present with the obstructive form due to a physical obstruction of the post-testicular genital tract, and >90% cases have normal spermatogenesis (2). NOA is the most severe form of male infertility, with an incidence of approximately 1% among adult males (3). The etiology of NOA is complex and involves environmental, genetic, epigenetic, and other factors (4). Testicular biopsy is the gold-standard diagnostic test for NOA. However, this diagnostic method may destroy focal spermatogenetic areas. Nevertheless, the potential treatment targets and mechanisms of NOA have not been clearly defined, limiting their suggestive and predictive roles in disease development and prognosis. Hence, further research is needed to better understand the mechanisms of spermatogenetic failure and identify biomarkers associated with NOA.

Autophagy is an evolutionarily conserved process that occurs under both physiological and pathological conditions of the body that is involved in the degradation and phagocytosis of damaged macromolecules and organelles to maintain cellular homeostasis and survival (5). Autophagy is associated with the pathogenesis of several types of diseases, such as infectious, cardiovascular, neurodegenerative, and autoimmune diseases and cancer. Importantly, autophagy also occurs in the male reproductive system and contributes to spermatogenesis by maintaining the homeostasis of germ cells, thus allowing to maintain normal development of the prostate gland under normal physiological conditions (6). Autophagy is regulated by several essential autophagy-related genes (ARGs), which participate in various stages of this process (7). ATG7, an ARG, has been recently reported to be highly expressed in NOA (5). In addition, Sha et al. (8) have demonstrated that autophagy-related cysteine peptidase family genes are associated with human spermatogenesis and identified ATG4D as a candidate gene that participates in NOA. However, these findings are insufficient for a detailed understanding of the molecular mechanisms underlying NOA.

Therefore, we aimed to identify ARGs associated with NOA and explore their molecular mechanisms using the Gene Expression Omnibus (GEO) database and Human Autophagy-dedicated Database (HADb).

## 2 Methods

In this study, the differentially expressed genes (DEGs) between the NOA and control groups were screened and intersected with ARGs to identify autophagy-related DEGs (ARDEGs). Subsequently, the ARDEGs were subjected to functional, protein–protein interaction (PPI) network, and functional similarity analyses. In addition, we performed immune infiltration analysis and constructed a hub gene–RNA-binding protein (RBP)–transcription factor (TF)–miRNA–drug network to explore the potential regulatory mechanisms of ARGs in NOA.

### 2.1 Data downloading and preprocessing

Two datasets associated with azoospermia were retrieved from the GEO database. GSE145467 (9) contained 20 testicular samples, 10 of which showed obstructive azoospermia and were considered to have normal spermatogenesis (control group). The other 10 samples showed NOA and were considered to have impaired spermatogenesis (azoospermia group). GSE45885 (10) contained 31 testicular biopsy samples, including 27 from patients with various types of NOA and impaired spermatogenesis (azoospermia group) and four with normal spermatogenesis (control group).

GSE145467 was downloaded from the GEO database using the GEOquery package version 2.54.1 (11) in R software (version 3.6.3; R Foundation for statistical computing, Vienna, Austria), and the probe that corresponded to multiple molecules was removed. The probe with the largest signal value was retained when multiple probes corresponded to the same molecule. For GSE45885, the expression matrices were directly downloaded from the GEO database, the probe corresponding to multiple molecules was removed, and the probes were not annotated to genes.

### 2.2 Differential expression analysis

For GSE145467, the limma package (12) version 3.42.2 was used for differential analysis between the two groups. For GSE45885, the difference between the two groups was analyzed using the GEO2R (13) online tool. Genes with logFC ≥ 1 and adjusted P < 0.05 were set as upregulated genes, and those with logFC ≤ -1 and adjusted P < 0.05 were set as downregulated genes. A volcano plot of the DEGs was plotted using the ggplot2 package (14).

### 2.3 Screening of autophagy-related DEGs

The list of ARGs was downloaded from the HADb. ARGs were then intersected with DEGs of GSE145467 and GSE45885, and the union of ARGs with the intersected genes was used to obatin ARDEGs. A heatmap was drawn using the ComplexHeatmap package (15) to show the differential expression of ARDEGs.

### 2.4 Correlation analysis and functional analysis of ARDEGs

The ggplot2 package was used to draw the correlation heatmap of ARDEGs in GSE145467 and GSE45885, and the Spearman statistical method was used for correlation analysis. The ClusterProfiler package (16) was used for Gene Ontology (GO) (17) (biological process [BP], molecular function [MF], and cellular component [CC]) and Kyoto Encyclopedia of Genes and Genomes (KEGG) (18) for enrichment analyses of ARDEGs. BH < 0.05 were considered statistically different, and all items were visualized as bubble graphs.

### 2.5 PPI network construction

Systematic analysis of the interactions between proteins in biological systems is important for understanding the working principle of proteins in biological systems, reaction mechanism of biological signals, and energy metabolism under special physiological states as well as the functional relationships between proteins (19). The STRING (20) database was used to build a PPI network of ARDEGs. Cytoscape (21) (version 3.8.0) was used to visualize the PPI network, and the hub gene was explored using the CytoHubba plug-in (22). The top 10 hub genes were screened based on the degree, closeness, and betweenness algorithms, and the intersection was visualized using a Venn diagram.

### 2.6 Summary of functional similarities analysis and chromosome distribution of hub genes

The GOSemSim software package (23) was used to evaluate the functional similarity between hub genes using geometric mean values of semantic similarity in BP, MF, and CC. The distribution of hub genes in the chromosomes was visualized using the Rcircos software package (24).

### 2.7 Estimation of immune infiltration in GSE145467

CIBERSORT (12) was used to estimate immune infiltration in GSE145467, and clustering of immune cells among samples was analyzed using principal component analysis (PCA). The correlation heatmap of immune cells in GSE145467 was drawn using ggplot2. The Spearman statistical method was used for correlation analysis. A box diagram was used to show the differences in immune cells in GSE145467.

### 2.8 Correlation analysis between hub genes and immune cell infiltration

The Spearman statistical method was used to analyze the correlation between hub genes and immune cell infiltration in GSE145467, which was visualized using a matrix heatmap. A scatter diagram was used to show hub genes–immune cells with correlation coefficients ≥0.8.

### 2.9 Assessment of DEGs at the single−cell transcriptional level

Human cell landscape database (http://bis.zju.edu.cn/HCL/) was used to visualise the expression patterns of some downregulated and upregulated genes in testis cell subgroups at the single-cell level. The selected cell samples contained different cell populations, including spermatogonial stem cell, differentiating spermatogonia, primary spermatocyte, round spermatid, elongated spermatid, macrophage, endothelial cell, myoid cell, sertoli cell, leydig cell and sperm.

### 2.10 Hub gene−RBP−TF−miRNA−drug prediction

The Encyclopedia of RNA Interactomesp (ENCORI) database collects data on miRNA–mRNA and miRNA–circRNA interactions from various sources such as software predictions, cross-linking and immunoprecipitation experiments, RNA–RNA interactome (25).. The TargetScan and miRanda algorithms in the ENCORI database were used to predict miRNAs targeting hub genes and construct the network.

hTFtarget integrates 7190 chromatin immunoprecipitation sequencing datasets of 659 TFs from the ENCODE, GEO, and SRA databases and high-confidence DNA-binding sequences of 699 TFs from the TRANSFAC, JASPAR, and HOCOMOCO databases. The regulatory capacity of TFs on target genes was quantified using an exponentially attenuated BETA model, and potential TF target genes were accurately predicted through epigenetic modification states on ROADMAP (26). Knock-TF is a comprehensive gene expression profile of the TF–gene knockout database (27). We performed TF prediction through the hTFtarget and KnockTF websites, and TFs predicted by Knock-TF were screened with logFC ≤ −1. The intersection of the predicted results was used to construct the network.

The RNA Interactome (RNAInter) database can simultaneously predict multiple molecules interacting with RNA, including >41 million RNA-related interactions involving >450,000 molecules, including RNA, protein, DNA, and compounds (28). We predicted RBP using the RNAInter database, and the RBP with a score of ≥0.3 was screened to construct the network.

The Drug-Gene Interaction Database (DGIdb) provides information regarding the association of genes with known or potential drugs (29). Data on drugs that interacted with hub genes were retrieved from the DGIdb.

### 2.11 Statistical analysis

Data were analyzed using R software (version 3.6.3). For comparison of two groups of continuous variables, the significance of normally distributed variables was estimated using the independent Student’s t-test and that of non-normally distributed variables was analyzed using the Mann–Whitney U test (Wilcoxon rank-sum test). Statistical significance was set at P < 0.05.

## 3 Results

### 3.1 Identification of DEGs

**Figure 1:**
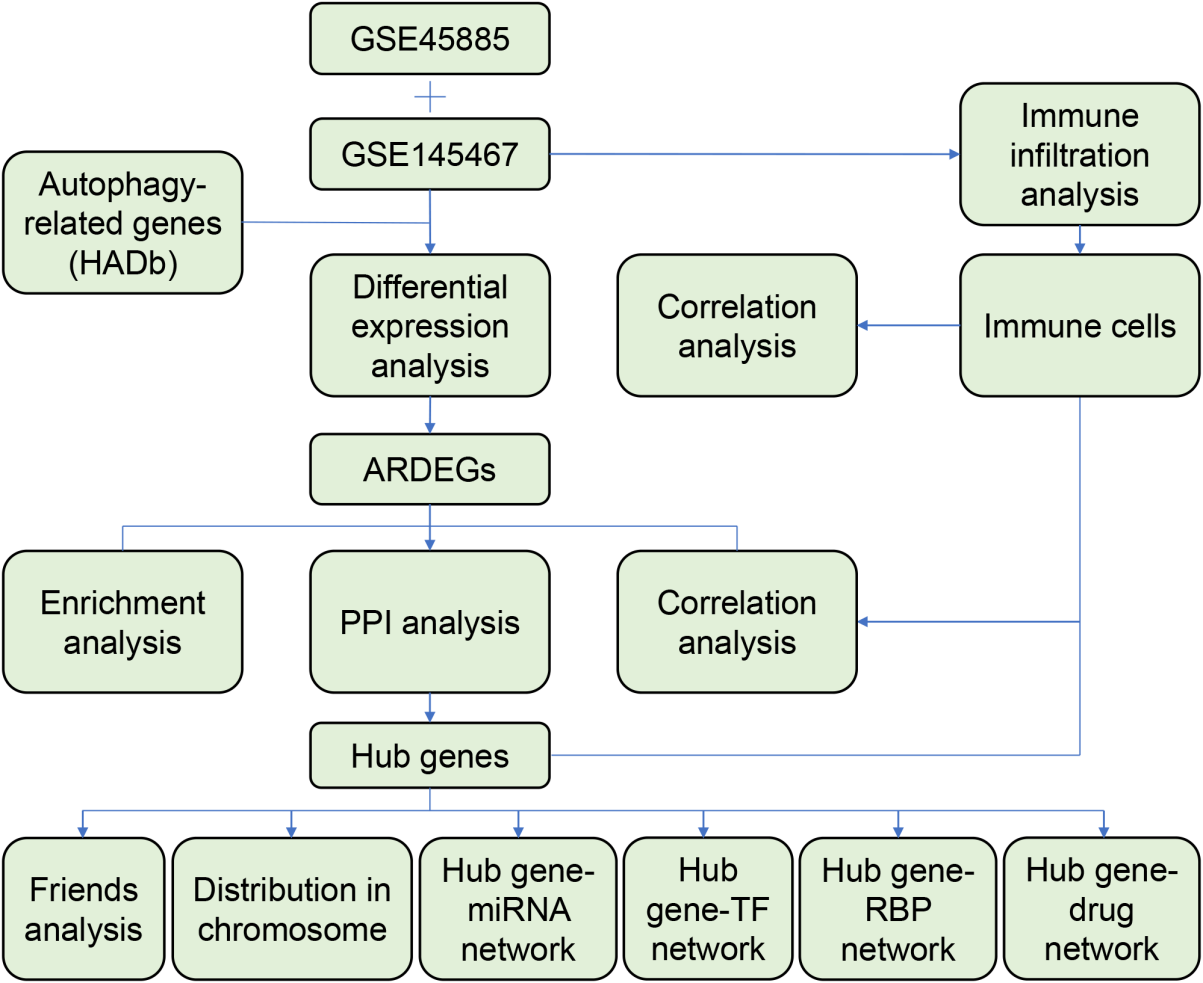
Flow chart of analysis.

**Figure 2:**
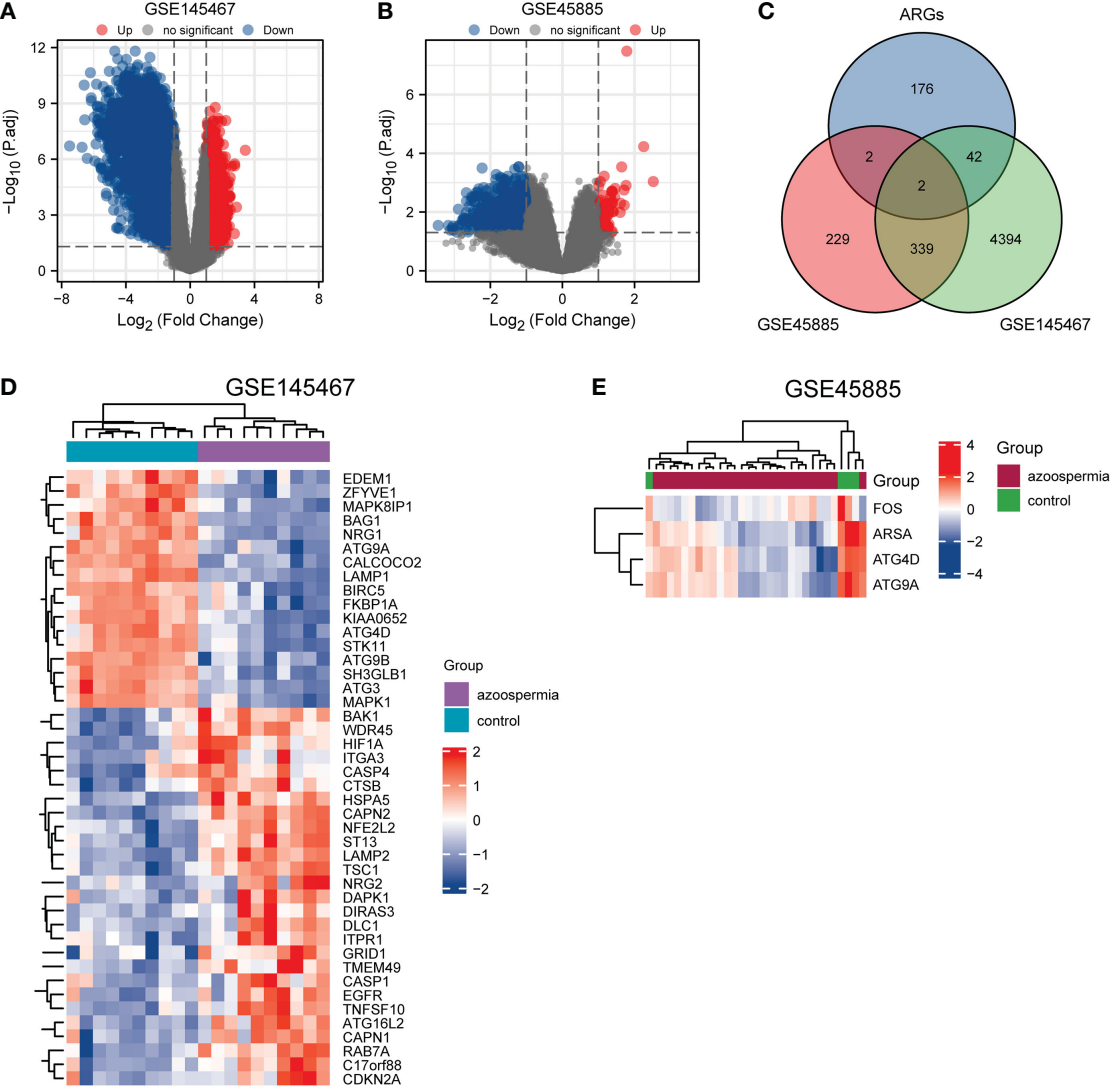
Identification of autophagy-related differentially expressed genes (ARDEGs). **(A)** and **(B)**: Volcano maps of differentially expressed genes () in GSE145467 and GSE45885, respectively. Abscissa is log2 fold change and ordinate is −log10 (P value). Red nodes represent upregulated differentially expressed genes, blue nodes represent downregulated differentially expressed genes, and gray nodes represent genes that are not significantly differentially expressed. **(C)**: Venn diagram of DEGs and autophagy-related genes. **(D)** and **(E)**: Heatmaps of ARDEGs in GSE145467 and GSE45885, respectively. Red represents high expression, and blue represents low expression.

**Figure 3:**
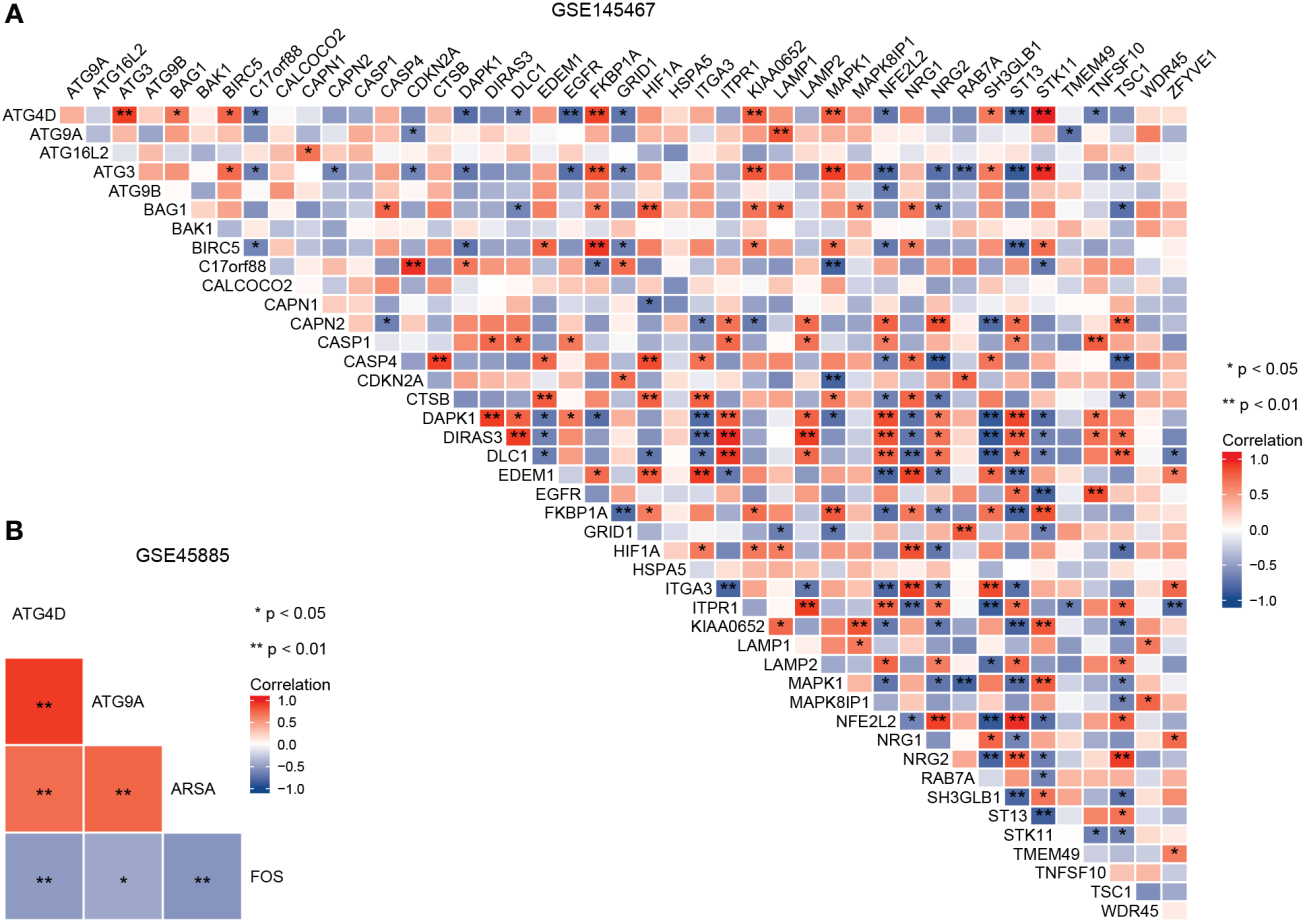
Correlation heatmap of autophagy-related differentially expressed genes in GSE145467 **(A)** and GSE45885 **(B)**.

A flowchart of the analysis is shown in **Figure 1**. We performed differential analysis of the GSE145467 and GSE45885 chip data. In total, 4777 DEGs were obtained in GSE145467, including 1605 upregulated and 1605 downregulated mRNAs. **Figure 2A** shows the volcano map of these DEGs. In total, 599 DEGs were obtained in GSE45885, including 75 upregulated and 524 downregulated mRNAs. **Figure 2B** shows the volcano map of these DEGs. Then, ARGs were obtained from the HADb, and the union of ARGs with the intersected DEGs in the two datasets was used to obtain 46 ARDEGs (**Figure 2C**). The expression of the 46 ARDEGs is shown in **Figures 2D, E**. The correlation heatmaps are shown in **Figures 3A, B**.

### 3.2 Functional enrichment analysis of ARDEGs

**Figure 4:**
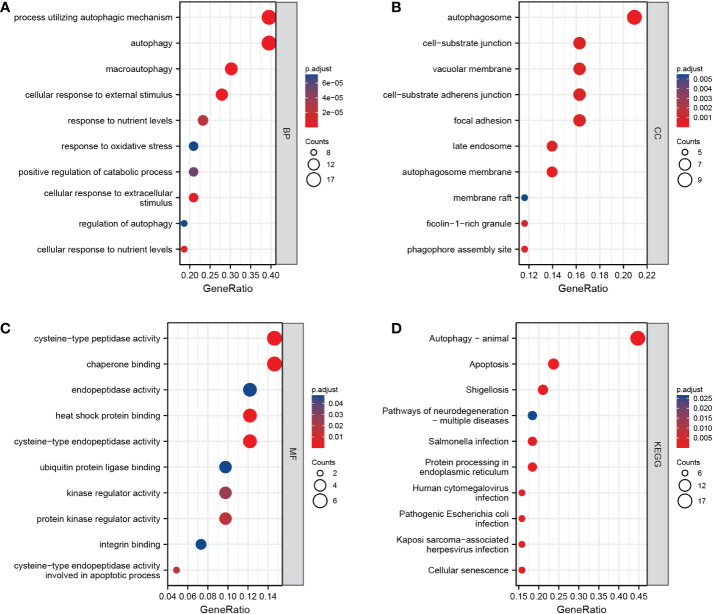
Results of enrichment analysis. **(A–C)**: Biological process, cellular component, and molecular function enrichment results for autophagy-related differentially expressed genes (ARDEGs). **(D)**: Kyoto Encyclopedia of Genes and Genomes (KEGG) enrichment results for ARDEGs. The abscissa is the number of enriched genes (gene ratio), and the ordinate is the Gene Ontology/KEGG terms.

We first converted the 46 ARDEGs into 43 Entrez IDs using the R package org.hs.eg. db (version 3.10.0). The 43 Entrez IDs were used for enrichment analysis. Under the condition of adjusted P < 0.05 and q < 0.2, we found 266 BP terms, 45 CC terms, 18 MF terms and 49 KEGG pathways (**Figures 4A–D**). ARDEGs were mainly enriched in BP terms such as autophagy, processes utilizing autophagic mechanisms, and macroautophagy. In addition, the pathways of autophagy, apoptosis, shigellosis, and protein processing in the endoplasmic reticulum were identified.

### 3.3 PPI network construction

**Figure 5:**
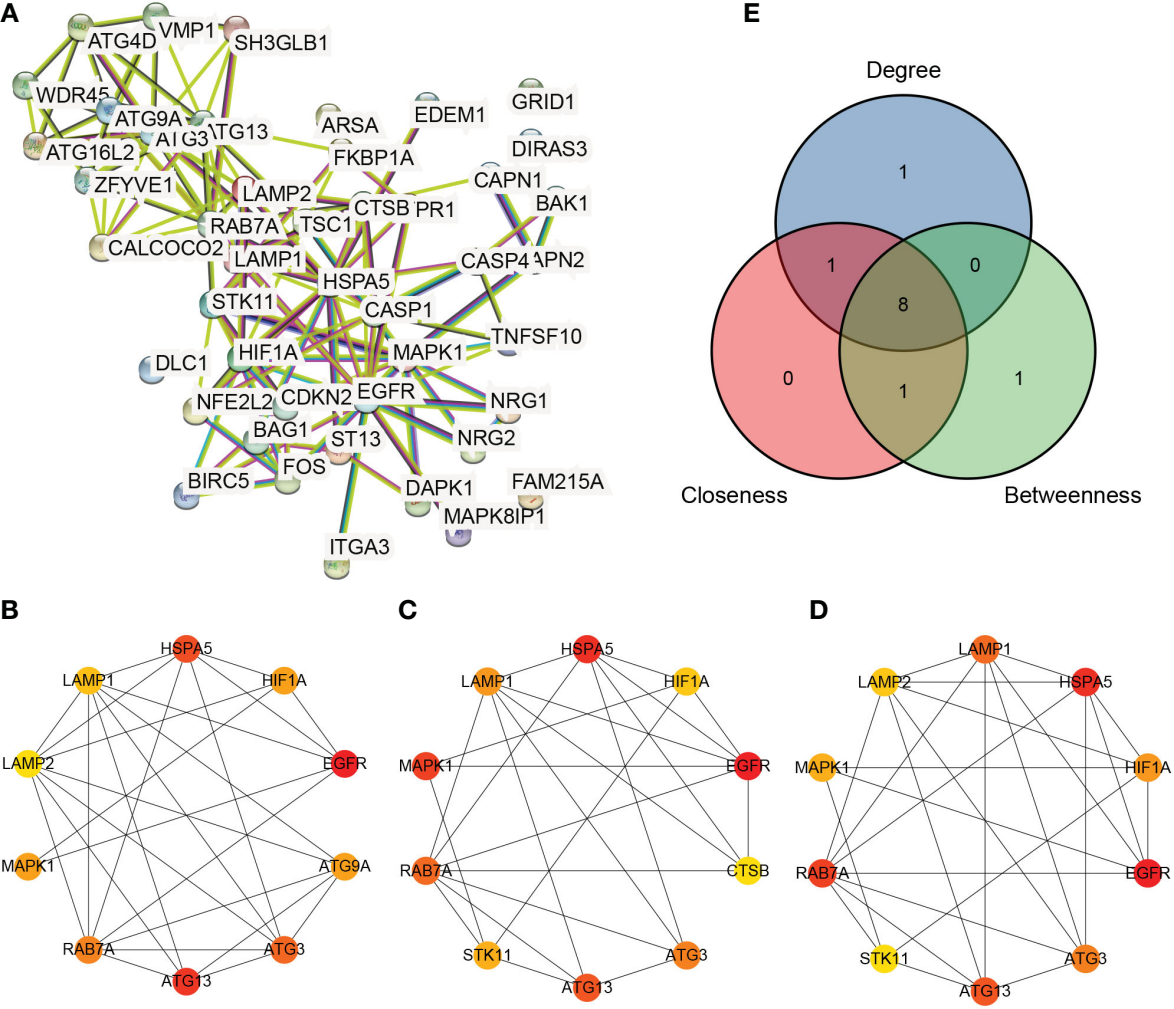
Protein–protein interaction network analysis. **(A)** Protein–protein interaction network of autophagy-related differentially expressed genes **(B–D)**: Hub genes calculated by the degree, betweenness, and closeness algorithms, respectively. **(E)**: Venn diagram of hub genes obtained using the three algorithms.

The ARDEG PPI network related to azoospermia was constructed according to the predicted results from the STRING database (**Figure 5A**). The results showed 45 ARDEGs and 129 PPI pairs in the network. The top 10 hub genes based on the three algorithms are shown in **Figures 5B–D**. Eight common hub genes (**Figure 5E**), including *EGFR*, *ATG13* (*KIAA0652*), *HSPA5*, *ATG3*, *RAB7A*, *HIF1A*, *MAPK1*, and *LAMP1*, were identified.

### 3.4 Expression, semantic similarity, and chromosome distribution of hub genes

**Figure 6:**
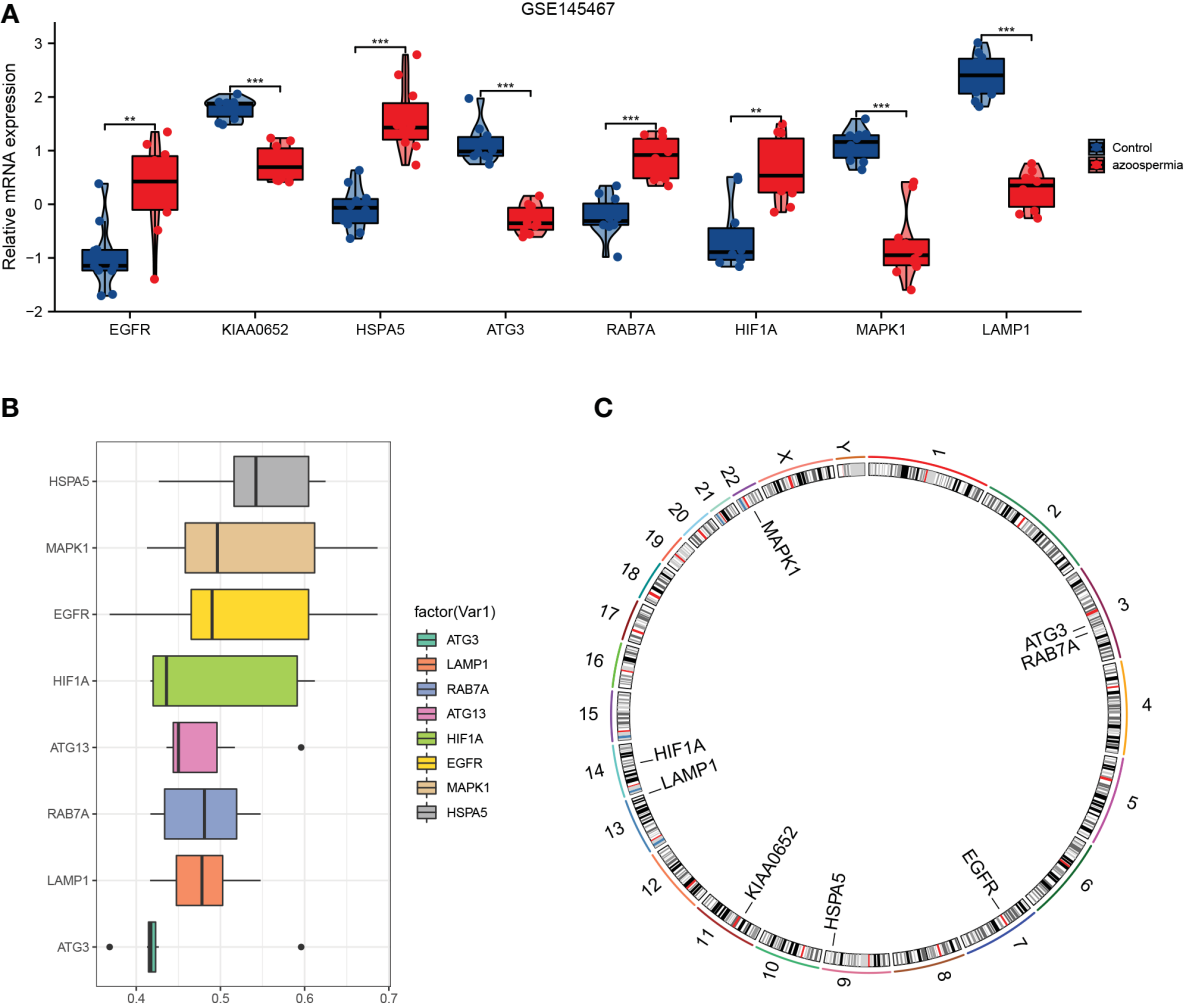
Expression **(A)**, semantic similarity **(B)**, and chromosome distribution **(C)** of hub genes. *P<0.05, **P<0.01, ***P<0.001.

Eight hub genes were identified in GSE145467 (**Figure 6A**). Functional similarity analysis showed that *HSPA5* might play a key role in azoospermia (**Figure 6B**). Analysis of the distribution of hub genes on the chromosomes revealed that *EGFR* was located on chromosome 7, *KIAA0652* on chromosome 11, *HSPA5* was located on chromosome 9, *ATG3* and *RAB7A* were located on chromosome 3, *HIF1A* was located on chromosome 14, *MAPK1* was located on chromosome 22, and *LAMP1* was located on chromosome 13 (**Figure 6C**).

### 3.5 Immune infiltration analysis in GSE145467

**Figure 7:**
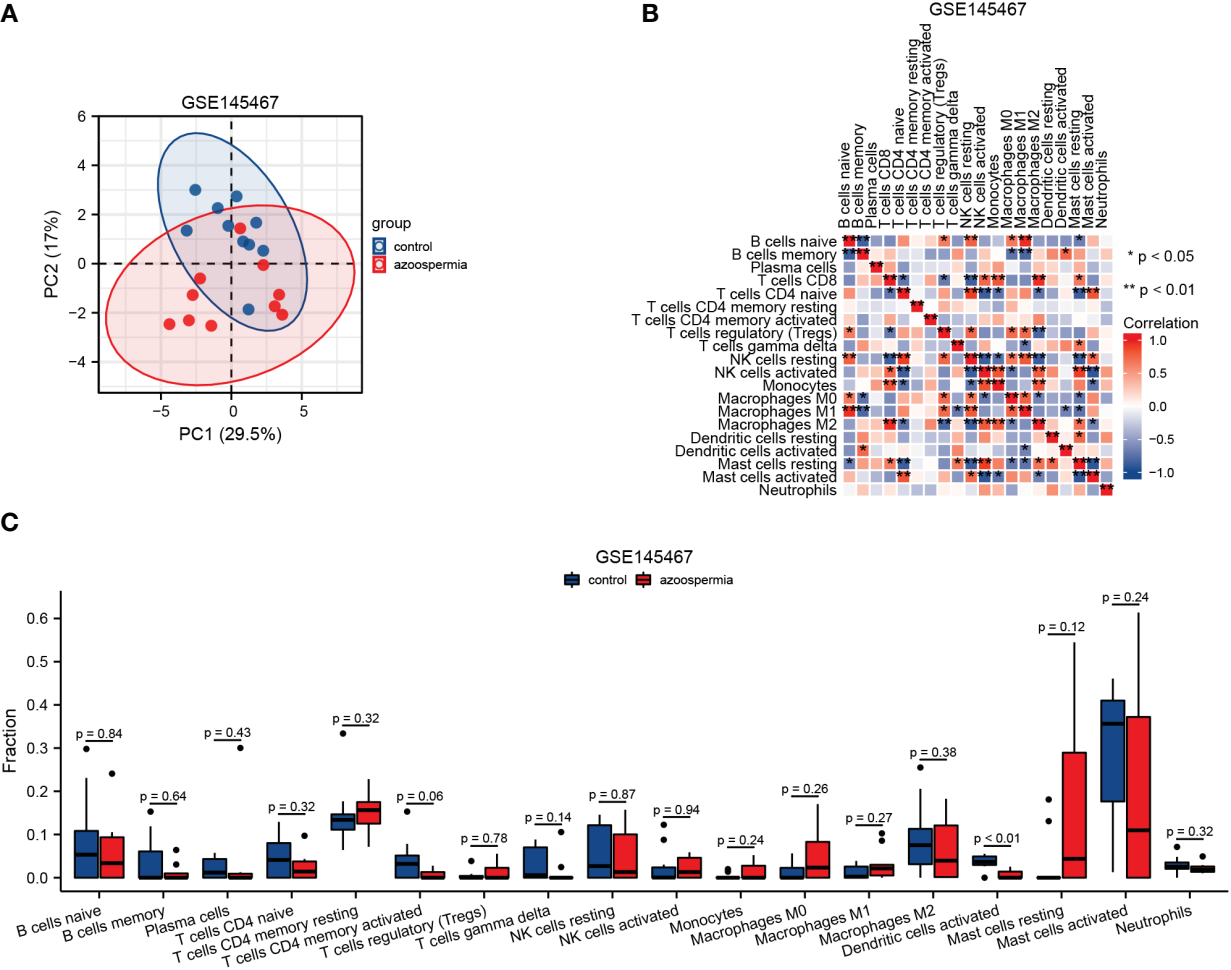
Infiltration of immune cells in GSE145467. **(A)**: Principal component analysis diagram of immune cell infiltration of samples from the normal spermatogenesis and impaired spermatogenesis groups. **(B)**: Immune correlation matrix. **(C)**: Differences in immune cells between the normal spermatogenesis and impaired spermatogenesis groups.

To further explore the relationship between hub genes and immune cell infiltration, we analyzed the immune cell infiltration spectrum in GSE145467 (**Figure 7**). The clustering of immune cells among samples in the azoospermia and control groups was demonstrated by PCA (**Figure 7A**). The immune correlation matrices are shown in **Figure 7B**. Among the 18 immune cell types involved, activated dendritic cells were significantly decreased in the azoospermia group compared to those in the control group (**Figure 7C**).

### 3.6 Correlation between hub genes and immune cell infiltration

**Figure 8:**
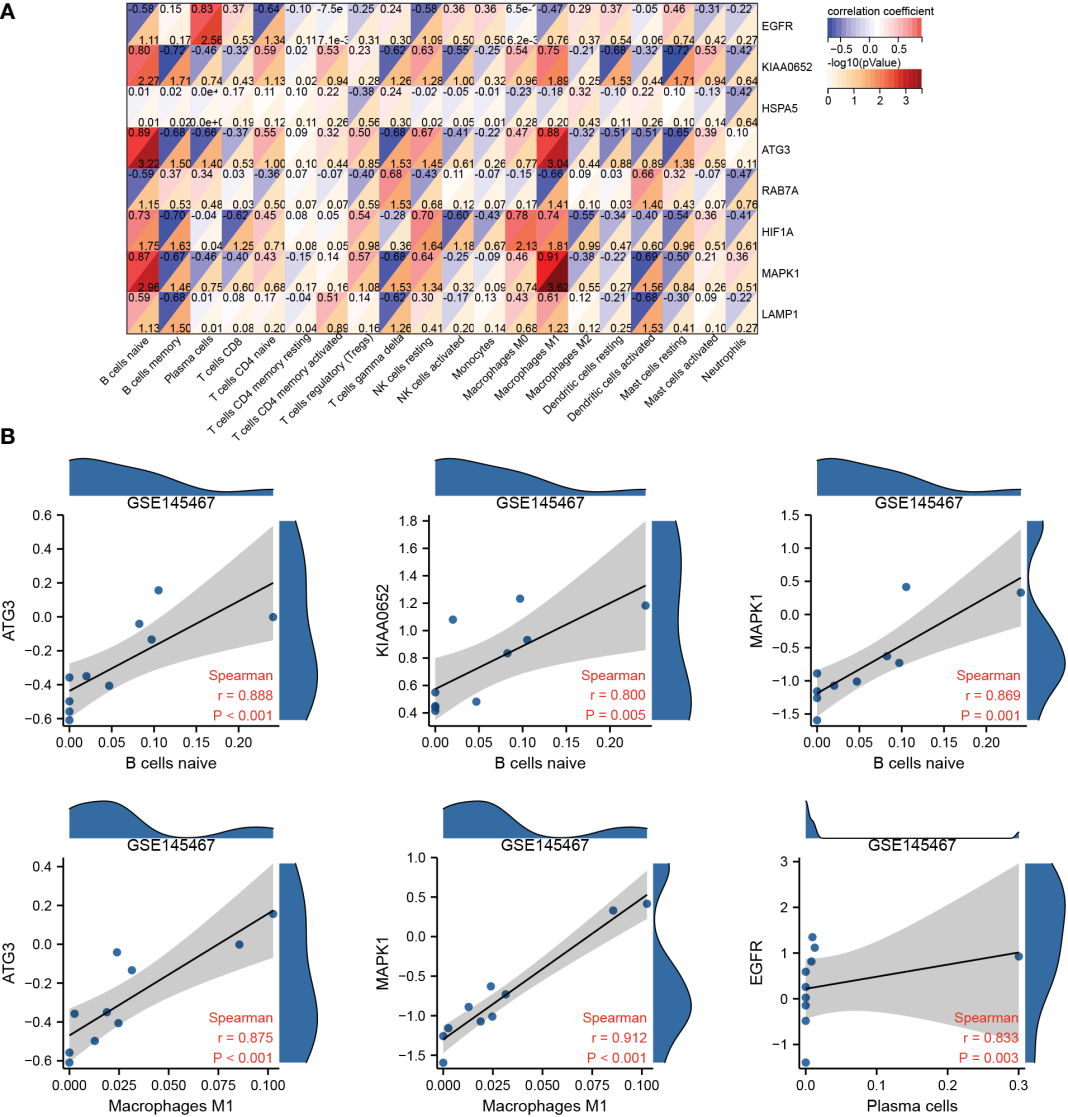
Correlation analysis of hub genes and immune cell infiltration. **(A)**: Correlation matrix between hub genes and immune cell infiltration in the samples of the impaired spermatogenesis group. **(B)**: Scatter plot of correlation between hub genes and immune cell infiltration in the samples of the impaired spermatogenesis group (only correlation coefficients ≥0.8 are shown).

Spearman correlation analysis revealed that hub genes in GSE145467 were strongly correlated with immune cell infiltration (**Figure 8A**), especially *ATG3* and naive B cells/M1 macrophages, *KIAA0652* and naive B cells, *MAPK1* and naive B cell/M1 macrophages, and *EGFR* and plasma cells. The correlation coefficients of the four pairs were >0.8 (**Figure 8B**).

### 3.7 Hub gene–miRNA–TF–RBP–drug network construction

**Figure 9:**
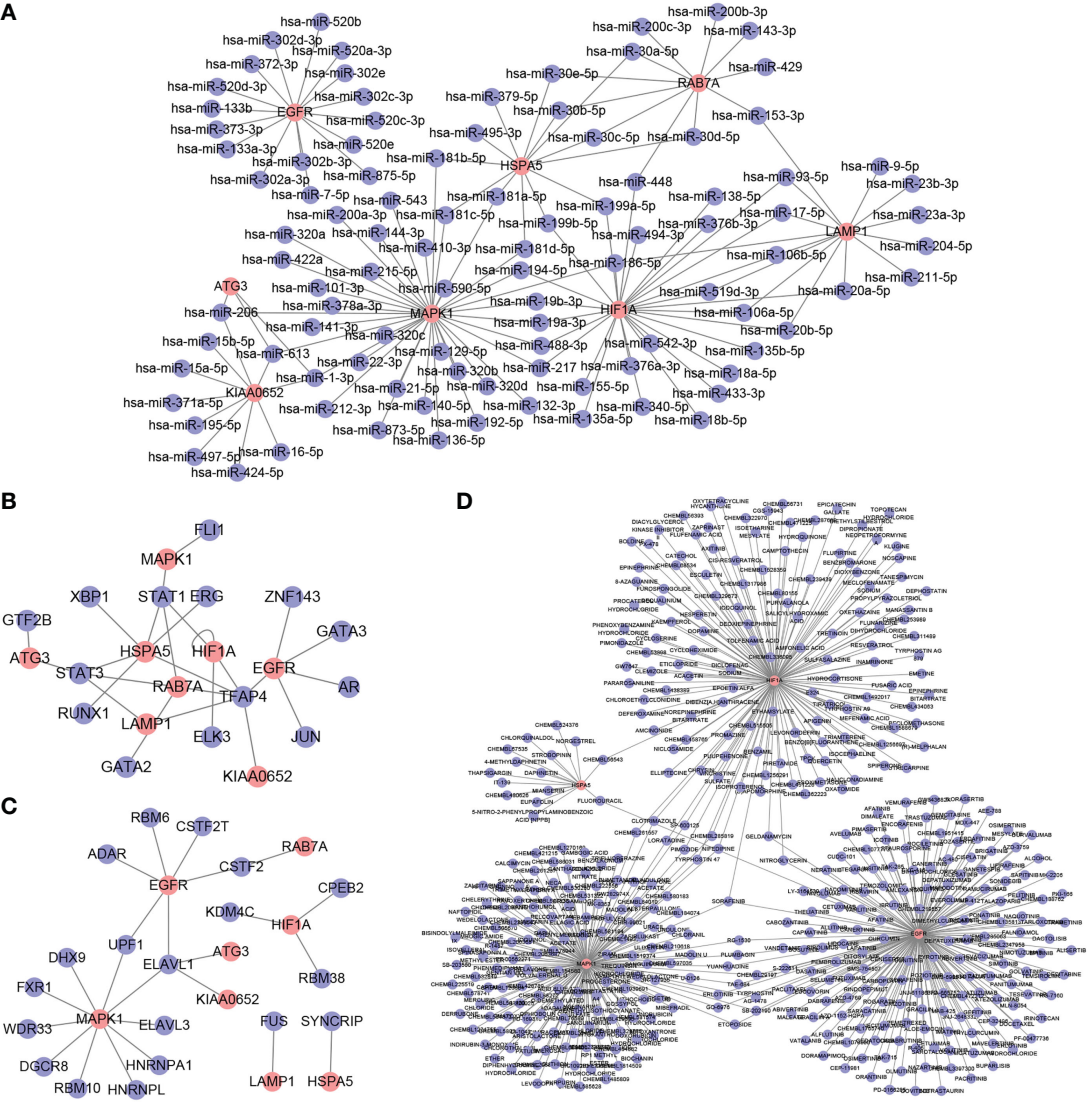
Hub gene–miRNA–transcription factor (TF)–RNA-binding protein (RBP)–drug network. **(A)**: Hub gene–miRNA network **(B)**: Hub gene–TF network **(C)**: Hub gene–RBP network **(D)**: Hub gene–drug network. The red nodes represent hub genes, and the blue nodes represent other molecules in the network.

To further explore the role of hub genes in the azoospermia molecular network, we predicted potential binding miRNAs, TFs, and RBP of hub genes as well as potential drugs targeting hub genes using open databases. miRNAs targeting hub genes were predicted using TargetScan and miRanda datasets from the ENCORI database, and hub gene-miRNA networks were constructed after intersection (**Figure 9A**). Combined with the hTFtarget and Knock-TF databases, TFAP4 was found to potentially transcriptionally activate *EGFR*, *KIAA0652*, *HSPA5*, *RAB7A*, *HIF1A*, and *LAMP1* (**Figure 9B**). The RNAInter predicted that ELAVL1 had the highest number of potential hub genes (**Figure 9C**). DGIdb showed that SORAFENIB could act on *EGFR*, *HIF1A*, and *MAPK1* (**Figure 9D**).

## 4 Discussion

NOA, which affects approximately 1% adult males, is a severe cause of male infertility (3). However, the pathogenesis of NOA is unclear. Autophagy is an evolutionarily conserved process that exists under both physiological and pathological conditions in the body (5) and influences spermatogenesis in the male reproductive system (6). Therefore, autophagy may have therapeutic potential in NOA; however, the underlying mechanisms need to be investigated. In this regard, identification of autophagy-related biomarkers in NOA may help improve the diagnosis and treatment of NOA.

In this study, 46 ARDEGs were identified between the azoospermia and control groups. These genes were involved in autophagy-associated functions and pathways. Eight hub genes were selected from the PPI network. Functional similarity analysis showed that *HSPA5* may play a key role in azoospermia. Immune infiltration analysis revealed that activated dendritic cells were significantly decreased in the azoospermia group compared to those in the control group. Hub genes, especially *ATG3*, *KIAA0652*, *MAPK1*, and *EGFR* were strongly correlated with immune cell infiltration. Finally, a hub gene–miRNA–TF–RBP–drug network was constructed.

The epithelial growth factor (EGF) family promotes meiotic initiation in neonatal mouse testes and responds to androgens (30). Strict regulation of EGF concentration in the testes is essential for spermatogenesis (31). EGF ligands function *via* EGFR, and EGFR-knockout mice exhibit severe spermatogenic defects (32). However, HSPA5 belongs to the HSP70 family and is expressed in the cytoplasm of both human spermatocytes and round spermatids. Additionally, HSPA5 may play an important role in the functioning of Sertoli cells and mature spermatozoa (33). Recently, YAPRAK et al. (34) demonstrated that the protein level of HSPA5 in spermatogenic cells was higher than that in non-spermatogenic cells isolated from testicular sperm extraction biopsies of patients with NOA and spermatogenic arrest. In the present study, functional analysis showed that *HSPA5* might play a critical role in azoospermia. Taken together, our results further confirm the key roles of *EGFR* and *HSPA5* in NOA. Concerning the other hub genes, there is no direct evidence of their associated with azoospermia or spermatogenesis.

In recent years, a growing body of evidence has demonstrated that the testicular immune microenvironment helps maintain normal spermatogenesis and immune privilege, while an abnormal immune microenvironment is closely related to azoospermia (35–37). Therefore, the present study investigated immune cell infiltration between the two groups as well as the correlation between hub genes and immune cell infiltration. As expected, activated dendritic cells were significantly decreased in the azoospermia group compared to those in the control group. Hub genes, especially *ATG3*, *ATG13* (*KIAA0652*), *MAPK1*, and *EGFR* were strongly correlated with immune cell infiltration. The results of single cell sequencing of *ATG3*, *ATG13* (*KIAA0652*), *MAPK1*, *and EGFR* in normal testicular tissue in the Human Cell Landscape also indicate that there are huge differences in their expression in different testicular tissue cells (**Supplementary Figure 1A-D**). This may suggest that the process of autophagy involves very complex spatial and temporal processes. Future collection of more single-cell sequencing data on testicular tissue of NOA patients will be more helpful to reveal the autophagy mechanism of NOA.

Dendritic cells are considered phagocytes involved in antigen presentation and induction of adaptive immunity (38). Recently, Duan et al. (39) found an inverse association between the abundance and phenotype of dendritic cells in the semen and sperm quality, suggesting that dendritic cell-mediated immune responses might contribute to male fertility disturbances. Consistent with the findings of the above study, our results showed that activated dendritic cells were significantly decreased in the azoospermia group compared to those in the control group. Furthermore, our results revealed significant correlations between *ATG3* and naive B cells/M1 macrophages, *KIAA0652* and B cells, *MAPK1* and naive B cells/M1 macrophages, and *EGFR* and plasma cells. Previous studies have described immune cells in normal testes and showed that testicular immune cells, including testicular macrophages and B cells, are associated with azoospermia (40, 41). Zhang et al. (42) have recently demonstrated that testicular macrophage polarization plays a critical role in the development of NOA. Given the important role of these immune cells in azoospermia, we speculated that *ATG3*, *KIAA0652*, and *MAPK1* might be implicated in azoospermia through the immune system.

To explore the potential regulatory mechanisms of hub genes in azoospermia, we predicted the potential binding miRNAs, TFs, and RBP of hub genes as well as potential drugs targeting hub genes using open databases. miRNAs have been suggested to play important roles in sperm defects, including azoospermia (43). For example, upregulation of miR-429 and miR-141 has been detected in idiopathic NOA using genome-wide miRNA expression profiling analysis (44). The two miRNAs were identified to regulate *RAB7A* and *MAPK1* in our study. The TF of TFAP4 was found to potentially transcriptionally activate six hub genes, including *KIAA0652*, *HSPA5*, and *RAB7A*, suggesting that TFAP4 might be involved in the development of NOA by regulating these hub genes.

Our study has some limitations. First, the sample size was small, and more microarray samples are needed to confirm the results, especially for NOA single-cell sequencing data. There are many differences between Single-Cell and Traditional RNA Sequencing Methods. In addition, the level of expression of selected characteristic genes at the single-cell level of the testes is very important, as some genes may represent important genetic information, such as reproductive toxicity (45). Second, there have been no experimental verifications to elucidate the functions of ARDEGs in NOA. Third, no clinical samples were collected for additional bioinformatics analyses aiming to clarify the molecular mechanisms underlying the development of NOA. Furthermore, the large number of datasets might have resulted in batch differences during analysis. Therefore, additional analyses with a larger sample size, including clinical samples, are needed to achieve more robust outcomes. More importantly, further experimental verifications are necessary to elucidate the biological functions of these predicted genes in NOA. Further *in vivo* and *in vitro* experiments may be employed to detect the diagnostic and therapeutic performance of these predicted target genes in NOA from our results. We would like to perform more careful examinations of the diagnosis and treatment effect of these predicted target genes in NOA by combing *in vivo* and *in vitro* techniques in future research.

In conclusion, eight hub genes, including *EGFR*, *HSPA5*, *ATG3*, *KIAA0652*, and *MAPK1* were implicated in azoospermia. These genes may serve as biomarkers for the diagnosis and treatment of this condition.

## Data availability statement

The datasets presented in this study can be found in online repositories. The names of the repository/repositories and accession number(s) can be found in the article/**Supplementary Material**.

## Author contributions

All authors contributed to the article and approved the submitted version.

## Supplementary material

Supplementary Figure 1 Gene expression in different types of cells within testis tissues, using single-cell RNA-seq data from Human cell landscape database. Expression of **(A)** ATG3, **(B)** ATG13, **(C)** MAPK1 and **(D)** EGFR for spermatogonial stem cell, differentiating spermatogonia, primary spermatocyte, round spermatid, elongated spermatid, macrophage, endothelial cell, myoid cell, sertoli cell, leydig cell and sperm.
