# Supplementary figures and images for: Integrative bioinformatics analysis to identify novel biomarkers associated with non-obstructive azoospermia
Source: Front Immunol. 2023 Mar 8;14:1088261. doi: 10.3389/fimmu.2023.1088261 (PMC10031032; doi:10.3389/fimmu.2023.1088261)

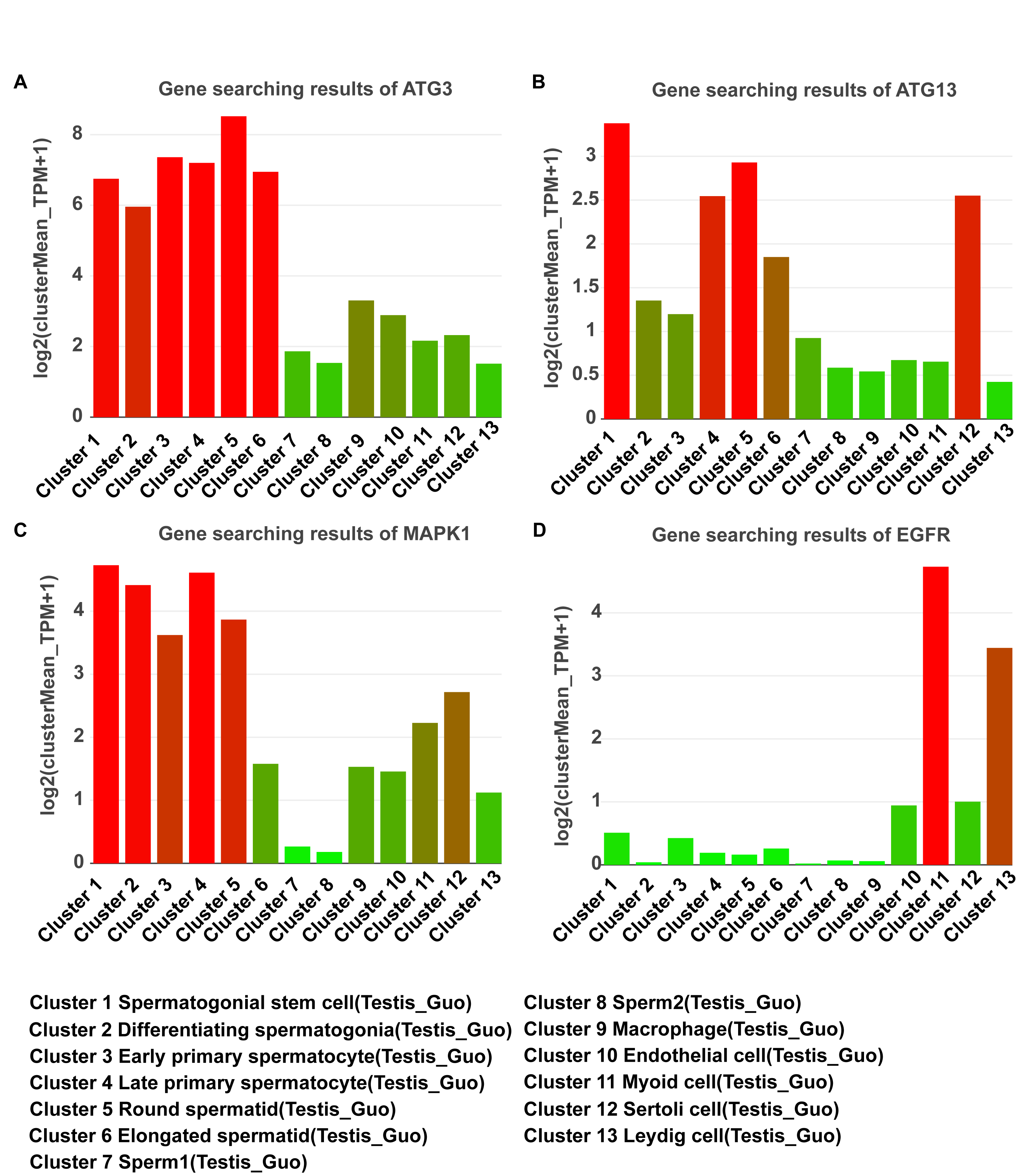

Supplement: Supplementary Figure 1 — Gene expression in different types of cells within testis tissues, using single-cell RNA-seq data from Human cell landscape database. Expression of (A) ATG3, (B) ATG13, (C) MAPK1 and (D) EGFR for spermatogonial stem cell, differentiating spermatogonia, primary spermatocyte, round spermatid, elongated spermatid, macrophage, endothelial cell, myoid cell, sertoli cell, leydig cell and sperm. [file Image_1.tif]
